# Effect of schizophrenia common variants on infant brain volumes: cross-sectional study in 207 term neonates in developing Human Connectome Project

**DOI:** 10.1038/s41398-023-02413-6

**Published:** 2023-04-10

**Authors:** Hai Le, Konstantina Dimitrakopoulou, Hamel Patel, Charles Curtis, Lucilio Cordero-Grande, A. David Edwards, Joseph Hajnal, Jacques-Donald Tournier, Maria Deprez, Harriet Cullen

**Affiliations:** 1 Centre for the Developing Brain, Perinatal Imaging and Health Department, King’s College London, London, UK; 2 Translational Bioinformatics Platform, NIHR Biomedical Research Centre, Guy’s and St. Thomas’ NHS Foundation Trust and King’s College London, London, UK; 3 NIHR Maudsley Biomedical Research Centre, King’s College London, London, UK; 4 Biomedical Image Technologies, ETSI Telecomunicación, Universidad Politécnica de Madrid & CIBER-BBN, ISCIII, Madrid, Spain; 5 Department of Medical and Molecular Genetics, King’s College London, London, UK

**Keywords:** Personalized medicine, Clinical genetics

## Abstract

Increasing lines of evidence suggest deviations from the normal early developmental trajectory could give rise to the onset of schizophrenia during adolescence and young adulthood, but few studies have investigated brain imaging changes associated with schizophrenia common variants in neonates. This study compared the brain volumes of both grey and white matter regions with schizophrenia polygenic risk scores (PRS) for 207 healthy term-born infants of European ancestry. Linear regression was used to estimate the relationship between PRS and brain volumes, with gestational age at birth, postmenstrual age at scan, ancestral principal components, sex and intracranial volumes as covariates. The schizophrenia PRS were negatively associated with the grey (*β* = −0.08, *p* = 4.2 × 10^−3^) and white (*β* = −0.13, *p* = 9.4 × 10^−3^) matter superior temporal gyrus volumes, white frontal lobe volume (*β* = −0.09, *p* = 1.5 × 10^−3^) and the total white matter volume (*β* = −0.062, *p* = 1.66 × 10^−2^). This result also remained robust when incorporating individuals of Asian ancestry. Explorative functional analysis of the schizophrenia risk variants associated with the right frontal lobe white matter volume found enrichment in neurodevelopmental pathways. This preliminary result suggests possible involvement of schizophrenia risk genes in early brain growth, and potential early life structural alterations long before the average age of onset of the disease.

## Introduction

Emerging evidence suggests schizophrenia may have neurodevelopmental origins [1], but the effect of schizophrenia common risk variants on normal early brain development in neonates remains unclear. For example, transcriptomic analyses have revealed high expression of schizophrenia risk genes during both foetal and perinatal brain growth [2, 3] and obstetric complications can increase incidence of the disease [4, 5]. Since the first two years of life is characterised by dynamic, regionally asynchronous and protracted growth of infant brain volume [6, 7], it is possible disruptions to this critical window either through genetic or environmental factors can set the brain on an unfavourable developmental trajectory [1, 8]. Preliminary magnetic resonance imaging (MRI) results in high-risk children of schizophrenic mothers and neonates with variants in psychiatric risk genes showed variations in brain tissue volumes and white matter connections [9–11], but such studies were few, and the results may not be disease specific.

Large scale MRI case-control studies of schizophrenia in adults and their meta-analyses have established widespread structural alterations in grey and white matter [12–14]. Compared to healthy controls, patients exhibited reduced global cortical surface area and cortical thickness changes across temporal, frontal, motor, somatosensory and parietal regions. Greater declines were coupled with longer illness duration and severity [12, 14, 15], and decreased structural integrity across multiple white matter tracts [16]. However, it is not apparent when these abnormalities arise during development [8] and whether the variability is influenced by schizophrenia-associated variants [17–19].

Despite the high heritability (80–85%), the genetic component of schizophrenia remains poorly understood [20]. A recent genome-wide association study (GWAS) of schizophrenia patients has identified 294 genome-wide significant single nucleotide polymorphisms (SNPs), primarily concentrated in genes prominent in neuronal and synaptic functions [21]. However, the additive effects of such SNPs as measured by polygenic risk scores (PRS) [22] explain less than 30% of the disease liability [21]. Although the current evidence linking structural brain imaging and schizophrenia PRS in adults remains inconclusive, with both positive and negative findings for subcortical nuclei [18, 19, 23], white matter microstructure [16, 23–26], total white matter volumes [27] and mean cortical thickness [28], the most consistent findings suggest aberrant interhemispheric and fronto-temporal connectivity may play a role in the aetiology of the disease [16]. This incomplete genetic penetrance and variable expressivity suggests schizophrenia and structural abnormalities observed is likely the result of complex interplay between multiple common variants of small effect and their interactions with the environment [29]. Therefore, linking disease-related phenotypes with genetic signatures of the disease in a healthy neonatal cohort may provide insights into its underlying early pathophysiology.

The aim of the current study was to examine whether brain volumetric changes associated with schizophrenia risk variants could be observed decades before the average age of onset of the disease, in a cohort of term-born neonates.

## Methods

### Cohort

The data used were analysed from infants, who were recruited from St. Thomas’ Hospital London, UK as part of the developing Human Connectome Project (dHCP) and had both genetic and imaging data processed by June 2019 (http://www.developingconnectome.org/data-release/second-data-release/) [30]. The dHCP was conducted according to the principles of the Declaration of Helsinki and ethical approval was given from the UK National Research Ethics Service. Written parental consent was provided for all subjects.

#### Genotyping and genetic quality control

The quality control for the genetics data used in this study is described in Cullen et al., 2020 [31]. Briefly, of the 628 saliva samples, genotype data were collected (Oragene DNA OG-250 kit) and genotyped for SNPs genome-wide on the Illumina Infinium Omni5-4 v1.2 array. For individuals with more than one sample, one sample was retained (randomly chosen). Individuals with genotyping completeness less than 95% were also excluded. Samples showing gender discrepancy or samples with genotyping failure of more than 1% of SNPs were removed. Further filtering was based on relatedness, and for every sample pair with relatedness above a cut-off (pi_hat ≥ 0.1875), one sample was retained (randomly chosen). SNPs being non-autosomal, having minor allele frequency ≤ 0.05, missing in >1% of individuals or deviating from Hardy-Weinberg equilibrium with a *p*-value < 1 × 10-5 were also filtered. This resulted in sample of 562 individuals with high-quality genetic data (4304505 SNPs).

#### Imaging data

**Fig. 1:**
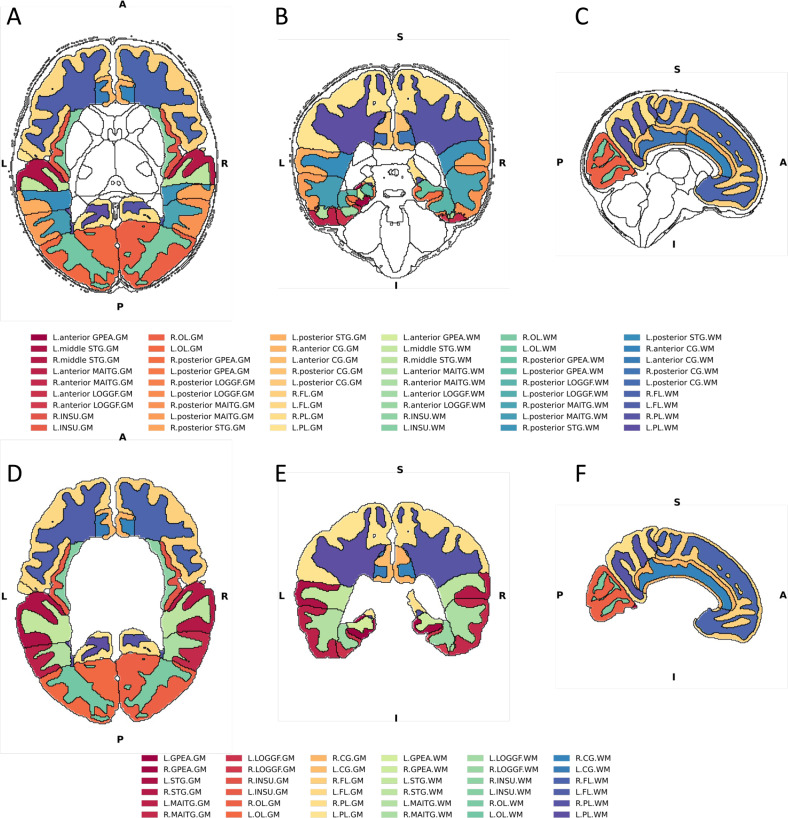
DrawEM neonatal brain segmentation. Visualisation of the brain regions examined in axial (**A**, **D**), coronal (**B**, **E**) and sagittal views (**C**, **F**). **A**–**C**—Black outline denotes segmented brain regions, where cortical white and grey matter regions and their subregions examined in this study are coloured. **D**–**F** Cortical white and grey matter regions examined in this study after summing up their respective subregions. GPEA gyri paraphippocampalis et ambiens, STG superior temporal gyrus, MAITG medial and inferior temporal gyrus, LOGGF lateral occipotemporal gyri, gyrus fusiform, CG cingulate gyrus, FL frontal lobe, PL parietal lobe, OL Occipital lobe, INSU Insula. Not shown anterior temporal lobe. L left side of the brain, R Right side of the brain. WM white matter, GM grey matter. A Anterior, P Posterior, S Superior, I Inferior, L Left, R Right.

Of the 675 individuals with imaging scans, 538 had both imaging and genotype data available [32]. Here, only term-born infants (born after at least 37 weeks of gestational age (GA)) were selected for further analysis (*n* = 422). Briefly, images were acquired on 3 T Philips Achieva scanner during natural sleep using a dedicated neonatal brain imaging system [30]. T2-weighted MRI were obtained using a TSE sequence with parameters TR = 12 s, TE = 156 ms and resolution (mm) 0.8 × 0.8 × 1.6. A combination of motion correction [33] and super-resolution reconstruction [34] techniques were then applied to the images to produce isotropic volumes of resolution (mm) 0.5 × 0.5 × 0.5. Subsequently, the T2 images were segmented with a DrawEM neonatal segmentation algorithm (https://github.com/MIRTK/DrawEM) presented in Makropoulous et al., 2014 [35]. This algorithm utilised spatial prior of 50 brain regions in form of 20 manually segmented atlases [36] in combination with tissue segmentation using Expectation-Maximisation technique to model intensities of different tissues classes and their subdivisions. This allowed the images to be accurately parcellated into 87 regions (Fig. 1A–C, Supplementary Table 1). The quality control was performed as a part of dHCP minimal processing pipeline [32]. The absolute volumes of each structure were measured as their total number of voxels multiplied by the voxel dimension [37].

### Data pre-processing

#### Volumetric data

From the original 87 segmented regions, we selected only those that are composed of cortex and subcortical white matter (Fig. 1A–C). Furthermore, volumes of subregions of the same brain structure in the same hemisphere were added up together. This resulted in 10 primary cortical regions (anterior temporal lobe, gyri parahippocampalis et ambiens, superior temporal gyrus, medial and inferior temporal gyri, lateral occipitotemporal gyrus, insula, occipital lobe, cingulate gyrus, frontal lobe and parietal lobe), each separated into 4 subregions (white matter and grey matter, left and right) (Fig. 1D, E, Supplementary Table 1). Total cortical grey and white matter volumes were calculated as the sum of the volumes of all examined cortical grey and white matter regions, respectively. The intracranial volume was computed as the sum of the volumes of brain tissue and cerebro-spinal fluid regions. Individuals with proportions of total cortical grey matter and total cortical white matter (grey matter volume or white matter volume divided by the total brain volume) over 3 standard deviations were considered outliers (4 individuals excluded).

#### Populations stratification

**Fig. 2:**
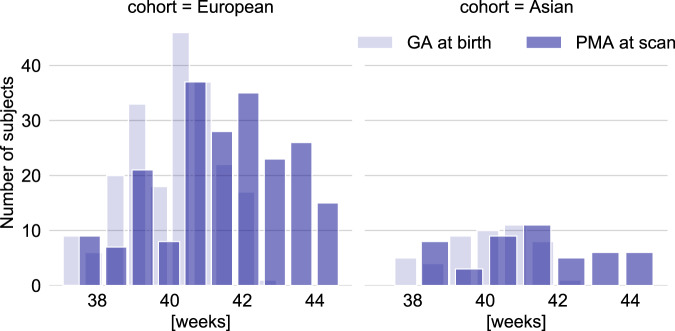
Term-born European and Asian neonatal cohorts. Gestational age at birth (GA) and postmenstrual age at scan (PMA) distribution across European and Asian cohorts.

Principal component analysis was performed on genetic data of each ancestral group, and visual examination of the components was used to exclude ancestral group outliers (12 individuals excluded). Since the discovery schizophrenia GWAS sample included primarily individuals of European (80%) and Asian (20%) ancestries, the ongoing analyses were carried out for the European (*n* = 207) and mixed (European and Asian ancestry combined; *n* = 257) cohorts (Fig. 2; Table 1).

**Table 1:** Demography of European and European and Asian (mixed) cohorts.

	European cohort	Mixed cohort
Individuals	207	257
Females/ Males	98/109	119/138
Mean age at birth (weeks)	40.07 (± 1.25)	40.06 (±1.24)
Mean age at scan (weeks)	41.50 (± 1.72)	41.49 (± 1.73)
Mean total brain volume (mm^3^)	372 792 (± 50 423)	370 496 (± 49 024)

#### Polygenic risk score

PRS were obtained for all individuals using odd ratios and *P*-values from summary statistics provided by the latest schizophrenia GWAS [21]. This analysis by the Schizophrenia Working Group of Psychiatric Genomics Consortium was performed on 67 390 people with schizophrenia and 94 015 controls. PRS were calculated utilising PRSice-2 [38] and estimated at 11 different *P*-value thresholds (P_T_): 10^−8^, 10^−7^, 10^−6^, 10^−5^, 0.0001, 0.001, 0.01, 0.05, 0.1, 0.5 and 1, such that each score was composed of only those SNPs with GWAS association *p*-value less than the respective threshold. Here, the 1000 Genomes project was used as the external LD reference panel [39]. PRS were computed as the sum of the risk alleles of an individual, weighted by odds ratio of the risk allele [40].

### Statistical analysis

For each dependent variable of interest, a univariate linear regression was performed with PRS as the independent variable, and ancestry principal components (first 3 or 5 components for the European ancestry and mixed ancestry cohorts, respectively), GA, postmenstrual age at scan (PMA), sex and intracranial volume (ICV) as covariates (i.e., X = PRS + Ancestry + GA + PMA + Sex+ ICV, where X is the dependent variable). The explained variance *R*^2^ due to the PRS is reported as the difference between the R^2^ of the full model with the PRS included as a covariate and the *R*^2^ of the null model without the PRS included as a covariate. This process was repeated for each of the 11 *P*-value thresholds.

Since both structural data and polygenic risk score thresholds are highly correlated, a method proposed by Li and Ji [41] to utilise eigenvalue variance (http://gump.qimr.edu.au/general/daleN/matSpDlite/) [42] was used to calculate the effective number of independent tests performed, M_eff_. This was computed independently for brain volumes (M_effBV_ = 13) and PRS (M_effPRS_ = 3). The multiple-comparison corrected *P*-value threshold was determined as *p* < 0.0013 (0.05 divided by the product of M_effPRS_ and M_effBV_). The main analysis was first performed on the European cohort due to the homogenous ancestry and repeated on the mixed (European-Asian) cohort.

Given our relatively small cohort, the results were further examined using three additional stability tests. Firstly, the sample was randomly divided into 2 equal data sets, and the regression analysis described above was carried out on both data sets separately. The results from both data sets were then compared for consistency. This test was repeated five times with different random splits in both European and mixed cohorts. Secondly, the sample PRS at the *P*-value threshold most associated with the brain volume is adjusted for ancestry components and converted to deciles. Here, the mean volumes of those brain regions reaching statistical significance adjusted for sex, GA, PMA, and ICV of individuals at the top and bottom 20% of the adjusted PRS were compared using *t*-tests. This test was performed in both European and mixed cohorts. Finally, since body weight has a large range in this dataset, individual birthweight was converted to Z-score (http://intergrowth21.ndog.ox.ac.uk/) and included as a covariate to the regression models.

### Gene-set enrichment analysis

To determine whether the SNPs contributing to the PRS at the *P*-value threshold most associated with infant brain volume also converged on relevant biological pathways, genes containing those SNPs were examined for their functions. Firstly, NCBI genes containing SNPs of interest within their protein-coding region as annotated in human genome build 37 (https://ctg.cncr.nl/software/magma) [43] were identified. To keep the gene list conservative, SNPs outside of the protein-coding region were not included. Secondly, to test for enrichment of biological function, the gene list was tested against 13 159 gene sets (pathways) obtained from MSigDB v.7.5.1 (curated canonical pathways from Reactome, KEGG and Wikipathways databases and curated Gene Ontology (GO) gene sets) [44] using a hypergeometric test, where for each pathway, the probability of randomly observing genes in the gene list was calculated [45]. This overrepresentation analysis was carried out using GENE2FUNC on FUMA platform [46] with 19,427 protein-coding genes from human genome Build 37 as background genes. Gene sets with adjusted Bonferroni *p*-value < 5.14 × 10^−8^ and with at least 5% of genes overlapping with our gene list were considered enriched. Finally, to further explore this result, we randomly selected 635 protein-coding SNPs from the same schizophrenia PRS SNP set at *P*-value threshold 0.05 and explored pathway enrichment in this random set. We repeated this process 10 times with different random sets and recorded the enriched pathways for each set. Additionally, other similar overrepresentation analysis software, including DAVID v.2021 (http://david.abcc.ncifcrf.gov/) [47, 48] and WebGestalt v. 2019 (http://www.webgestalt.org/#) [49] were employed to confirm the FUMA findings. We performed this functional analysis for the SNP set most associated with the imaging phenotype in the European cohort.

## Results

### Reduced volume in frontal and temporal regions associated with high PRS

**Fig. 3:**
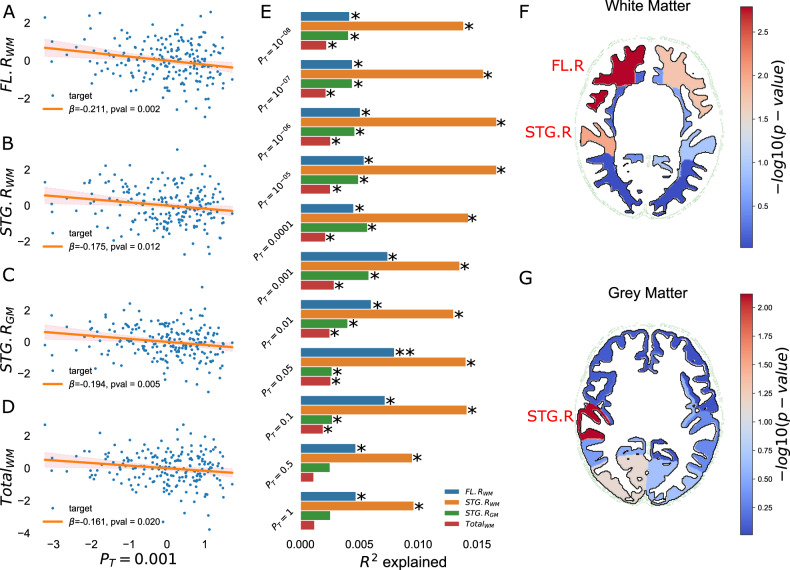
Visualisation of the significant association between the schizophrenia PRS and the regions of interest in the European ancestry cohort (*n* = 207). **A**–**D** Scatter plots of infant brain volume against Schizophrenia PRS at *P*_T_ = 0.001. Brain volumes have been adjusted for GA, PMA, sex and ICV. PRS have been adjusted for the first 3 ancestral principal components. Axes are standardised to zero mean and unit variance. The shaded area denotes the confidence interval (**E**): Bar plot of brain volume variance *R*^2^ explained by PRS, calculated as the difference between the *R*^2^ of the full model with PRS as a covariate and that of the null model without the PRS as a covariate. *nominal significant result; **result surviving multiple testing correction. **F**, **G** Visualisation of the significant associations between the PRS at *P*_T_ = 0.001 and brain volumes of white (**F**) and grey matter (**G**) regions of interests. Colour bar denotes the range of –log_10_(*p*-value). (FL.R- right frontal lobe, STG.R- right superior temporal gyrus).

We found a statistically significant negative association between PRS and the right frontal lobe white matter (FL.R_WM_) at *P*_T_ = 0.05 in the European-ancestry cohort (*R*^2^ = 0.0088, *β* = −0.0973, *p* = 0.0012). Nominally (*p* < 0.05) significant associations were also found consistently across multiple PRS *P*-value thresholds with the right superior temporal gyrus grey matter (STG.R_GM_), the right superior temporal gyrus white matter (STG.R_WM_), and the total white matter. The directions of all reported associations were as expected: greater genetic risk scores were associated with smaller grey and white matter volume (Fig. 3, Supplementary Table 2).

Repeating our main analysis in the European and Asian ancestry cohort, we found robust associations in the same directions in the same brain regions (Supplementary Table 3; Supplementary Figure 1). Here, the association between PRS and the right superior temporal gyrus grey matter volume survived multiple testing correction at three *P*-value thresholds (highest R^2^ = 0.0068, *β* = −0.0866, *p*-value = 0.0011 at *P*_T_ = 0.001), between PRS and the right superior temporal gyrus white matter at seven *P*-value thresholds (highest *R*^2^ = 0.0205, *β* = −0.1751, *p*-value = 0.0008 at *P*_T_ = 0.05) and between PRS and the right frontal lobe white matter at four *P*-value thresholds (highest *R*^2^ = 0.01, *β* = −0.105, *p*-value = 0.0001 at *P*_T_ = 0.001). Performing stability tests by halving the samples or comparing between the top- and bottom 20% showed consistent associations in the same brain regions and PRS in both cohorts (Supplementary Table 4 and 5). Finally, including birth weight as covariates in the regression models showed similar directions of associations in the same brain regions and PRS thresholds in both cohorts (Supplementary Table 6A, B).

### Overlapping SNPs involved in neurodevelopmental function

Functional analysis of SNPs underlying our most robust association in the European ancestry cohort, namely the schizophrenia risk score at *P*-value threshold 0.05 and the right frontal lobe white matter, found enrichment in neuron development pathways. Of the 29 942 SNPs contributing to the risk score at *P*-value threshold 0.05, 1458 were nominally associated (*p* < 0.05) with the right frontal lobe white matter in linear regression (Brain Volume ~ Risk allele frequency + Ancestry + GA + PMA + Sex + ICV). This reduced set of SNPs was reasoned to represent a selection of SNPs associated with increased risk for schizophrenia and right frontal lobe white matter in our neonatal cohort.

**Fig. 4:**
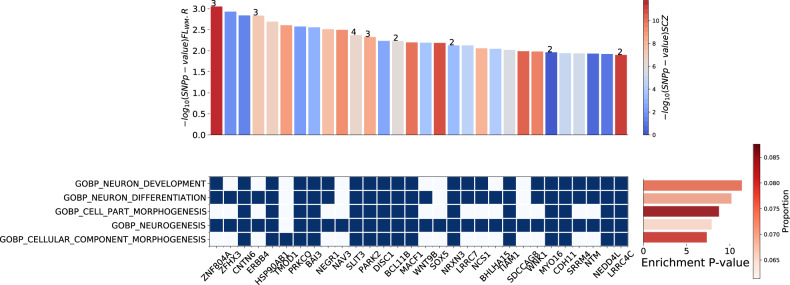
Top five enriched pathways and their corresponding gene lists as calculated by FUMA [46]. Top: bar plot of –log(*p*-value) of SNPs mapped to genes in gene list ordered by their association with the right frontal lobe white matter in the European cohort. Numbers on the bar denote the number of SNPs if more than 1 SNP associated with right frontal lobe white matter is mapped to that gene. Colours in the bar plot denote the –log(*p*-value) of the SNPs (the most associated SNP if there are more than 1 for that corresponding gene) with schizophrenia as calculated by schizophrenia GWAS [21]. Bottom: Enriched gene sets and their overlapping gene list (shown are the top 30 genes ordered by their *p*-value association with the right frontal lobe white matter). Blue square- the gene in the gene list is present in that pathway. Left: bar plot of the gene-set enrichment adjusted *p*-value. Colours denote the proportion of genes in the gene set found in the gene list. GO_BP – Gene Ontology Biological Processes.

Of the 1458 SNPs, 635 are found in protein coding regions of 550 genes (Supplementary Table 7). Overrepresentation analysis revealed 157 genes were significantly enriched in several Gene Ontology biological processes and cellular component terms (Fig. 4; Supplementary Table 8A), the most prevalent of which were neuron development (Gene Ontology biological processes term 0048666: *p* = 2.12 × 10^−16^), neuron differentiation (Gene Ontology biological processes term 0030182: *p* = 3.42 ×10^−15^) and cell part morphogenesis (Gene Ontology biological processes term 0032990: *p* = 9.44 × 10^−14^). Multiple gene-set analysis rerun with random set of 635 protein coding SNPs from the same 29 942 schizophrenia SNPs did not show the same enrichment of neuron differentiation and neuron developmental pathways (Supplementary Table 8B). Finally, analysis with DAVID v2021 and WebGestalt v2019 yielded similar results. (Supplementary Table 9).

## Discussion

We identified evidence of early volumetric changes associated with schizophrenia PRS in the cortical grey and white matter regions. Directions of the associations were consistent with previous findings in adult schizophrenia patients, where greater risk of the disease was associated with reduced brain volumes. The results for the European and the combined European and Asian cohorts were consistent, indicating common genetic variation associated with schizophrenia risk may have similar effects on infant brain volumes across these ethnicities. The schizophrenia risk variants associated with the structural alterations also appear to be enriched for early brain organisation processes, suggesting schizophrenia pathophysiology may have a neurodevelopmental origin.

### Clinical relevance of neuroimaging observations with pathophysiology of schizophrenia

Structural abnormalities in the grey matter superior temporal gyrus have been consistently linked to schizophrenia [50, 51] and positive symptoms of schizophrenia such as auditory hallucinations and thought disturbances [52]. In later adulthood, patients can exhibit steeper volumetric reduction [53], decreased cortical thickness and reduced neural activity in this region [52]. Similarly, progressive declines in total white matter, frontal and superior temporal white matter volumes have all been previously found in schizophrenia patients [14] and appear to advance with age after the disease onset irrespective of other risk factors such as sex, family history or perinatal risk [54]. Decrements in both frontal and temporal white matter volumes have also been associated with poorer executive functioning [55], with the frontal volume loss associated with greater negative symptom severity. Finally, it is possible the predominant right hemisphere associations found here may reflect the asymmetrically lateralised development of the prefrontal and temporal lobes most pronounced during the foetal period [56].

### Comparison with previous neonatal and adult PRS imaging studies

The results presented here also contribute to the small body of literature of schizophrenia research in neonates. Gilmore et al. [57]. reported no group differences in grey and white matter volumes in a sample of 26 high-risk infants, born to mothers with schizophrenia and their matched controls. Recent work by Knickmeyer et al. [10]. and Cullen et al. [11]. have revealed common risk variants in psychiatric risk genes may be associated with the volume of grey matter in frontal, temporal and subcortical structures in preterm and high-risk infants. Nevertheless, these findings are not specific to schizophrenia.

Despite the well-established structural alterations associated with the disease, exploring the association between schizophrenia PRS and neuroimaging phenotypes has yielded mixed results. Consistent with our findings are the negative associations between PRS and the total white matter volume [27, 58] and grey matter superior temporal gyrus [59] found in adults, although they have not been well replicated in larger independent samples [23, 60–62]. The lack of reproducible results could be for many reasons, including possible small genetic overlap between brain phenotypes examined and schizophrenia (e.g., subcortical structures [18, 19]). However, it could also be due to variation in the disease diagnosis, highly distinct genetic determinants for structural variability at different ages [63] and pleiotropic effects of such variants on imaging phenotypes and the disease. Development of other more focused methodologies to identify shared cross-trait genetic architecture [64] or examination of longitudinal change of phenotypes instead of cross-sectional differences may be needed.

Finally, we also explored a relationship between schizophrenia PRS and familial risk of psychiatric disease using a mental health questionnaire but found no association (full details are provided in Supplementary Information 1).

### Shared schizophrenia genetic risk liability between European and Asian cohorts

Recent examination of genetic components of schizophrenia in European and East Asian individuals revealed high similarity in common variants, indicating that the genetic basis of the disease and its biology may be shared broadly across populations [65, 66]. Indeed, despite population differences in genome-wide allele frequencies and patterns of linkage disequilibrium [67], evidence suggests that at least some degree of schizophrenia risk variants was shared across ethnic groups prior to human diaspora out of Africa [68]. As the current PRS was derived from the discovery GWAS sample of patients of both European (80%) and East Asian (20%) ancestry, the robust associations with infant brain volumes found in both the homogenous European and the heterogeneous mixed European and Asian cohort suggest future inclusion of more diverse population in GWAS would be beneficial [21].

### Exploratory analysis of gene sets

By selecting SNPs associated with the schizophrenia and infant right frontal lobe white matter volume in our cohort, we identified genes overrepresented for pathways involved in neuron development, many of which have previously been validated in schizophrenia models (e.g., *ZNF804A* [69], *DISC1* [70], *NRXN3* [71]). Interestingly, our results are also consistent with previous GWAS findings, where common variants associated with neonatal brain volumes were located in genes involved in neurogenesis, neuronal migration and differentiation [63]. Whilst our functional analysis focused exclusively on SNPs within the protein coding regions, it is important to note that most SNPs are found in non-protein coding regions and can influence expression of nearby genes [72]. Furthermore, functional clustering methods should be regarded as exploratory, as the analyses are biased toward larger well-studied processes [45]. In addition, many of the genes are also linked to other neurodevelopmental disorders including autism spectrum disorder and intellectual disability and have been shown to induce alterations in white matter [73]. Together, the results support the neurodevelopmental models of schizophrenia [8], and pathology of the disease seen later in life may be a consequence of early perinatal insults [74]. However, the current findings cannot provide evidence for or against other hypotheses of the disorder aetiology (e.g., two hit models or neurodegenerative models), as more focused and testable hypothesis may be needed [8].

### Limitations

The main limitations in our study lie in the small sample size and simplicity of PRS. Therefore, the result presented here must be considered preliminary and additional independent sample must be used to confirm the findings. Nevertheless, the dataset examined is unique in its combination of genetic and detailed neuroimaging data at birth and adequate approaches have been taken to improve interpretability of the results. Here, only term-born infants of European ancestry (and Asian ancestry in the mixed cohort) were selected, due to (1) overwhelming presence of European population in the discovery GWAS (80% European and 20% Asian), which can impact the interpretability of the PRS and (2) known impact of prematurity on imaging phenotypes. Although incorporating the Asian cohort may have introduced additional heterogeneity, the consistency of results across the European and larger mixed cohorts was reassuring. Finally, while the simplicity of the PRS means it cannot readily provide biological interpretation of the findings, the enrichment analysis may provide relevant targets for further research.

## Conclusions

In summary, our study reports negative associations between the PRS with the grey and white matter volumes in the superior temporal gyrus, the white matter volume in the frontal lobe and the total white matter volume in a cohort of term neonates. The result can provide further guidance for future work studying the origins of schizophrenia and its trajectory across the lifespan.

## Supplementary information

Supplementary Figure 1

Supplementary Figure 1 legend

Supplementary Information 1

Supplementary Table 1

Supplementary Table 2

Supplementary Table 3

Supplementary Table 4

Supplementary Table 5

Supplementary Table 6A

Supplementary Table 6B

Supplementary Table 7

Supplementary Table 8

Supplementary Table 9

## Data Availability

Statistical analysis was performed using *statsmodels* module v 0.13.2 (https://www.statsmodels.org/stable/index.html) in Python. The MRI data used is freely available: https://biomedia.github.io/dHCP-release-notes/. Code used to perform statistical analysis in this study is available on https://github.com/lehai-ml/nimagen.
