# Supplementary figures and images for: Effect of schizophrenia common variants on infant brain volumes: cross-sectional study in 207 term neonates in developing Human Connectome Project
Source: Transl Psychiatry. 2023 Apr 10;13:121. doi: 10.1038/s41398-023-02413-6 (PMC10085987; doi:10.1038/s41398-023-02413-6)

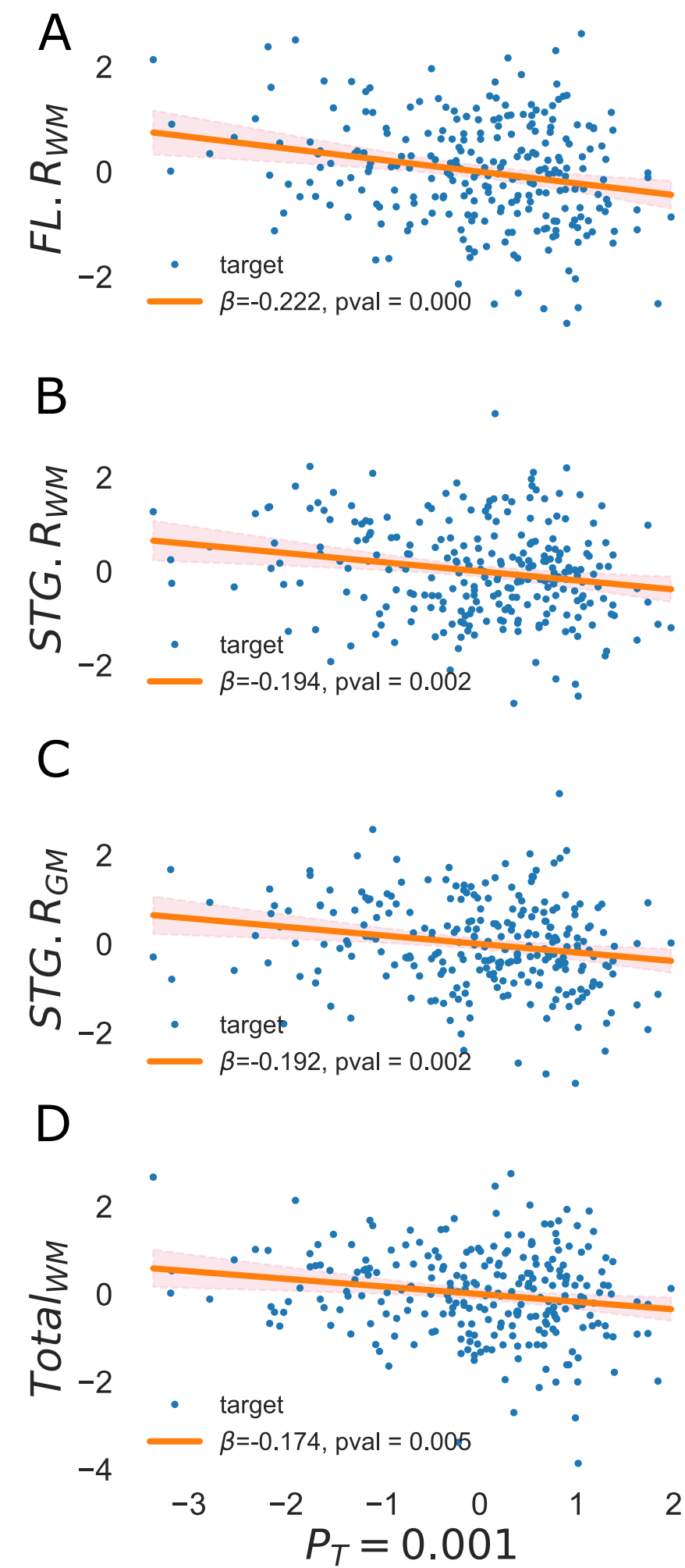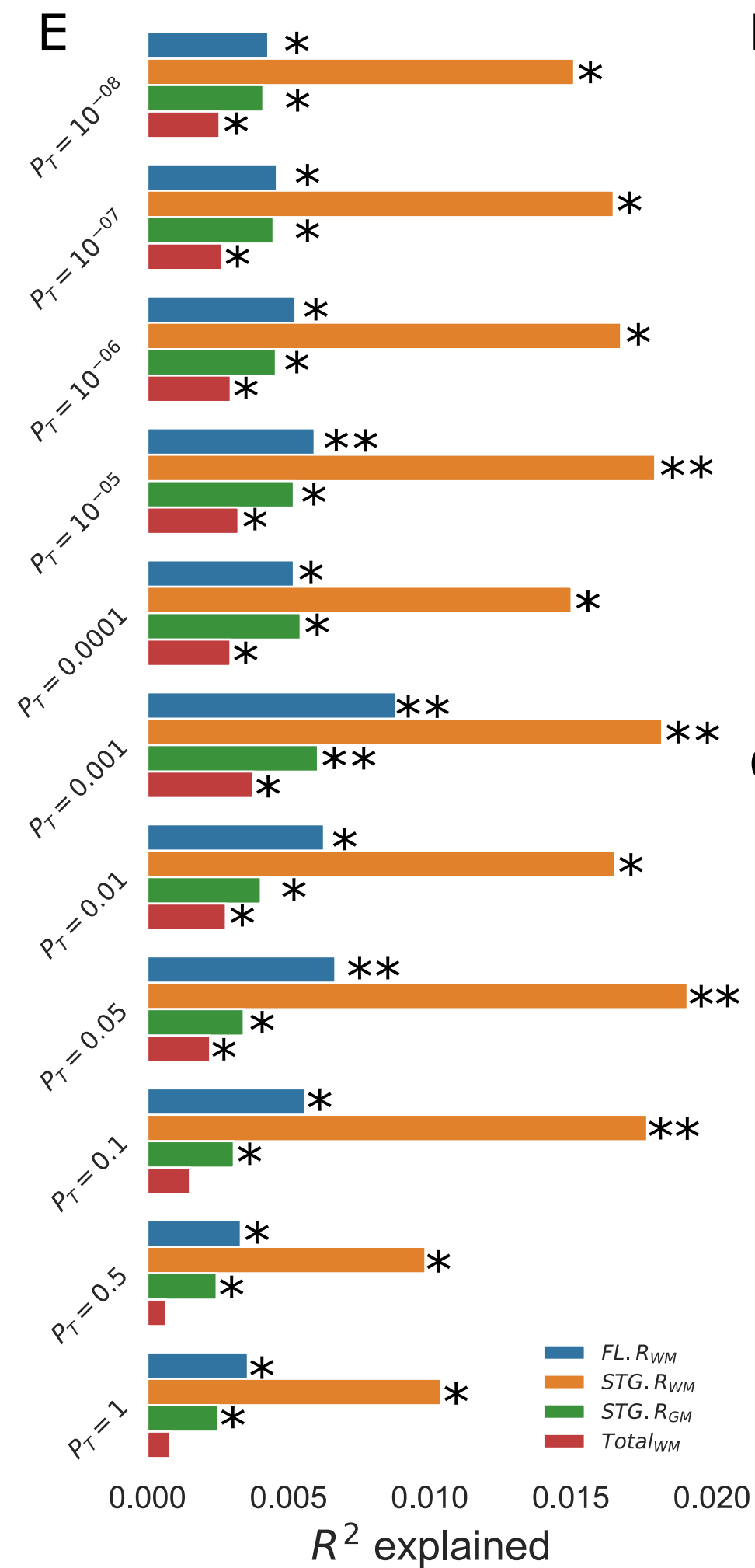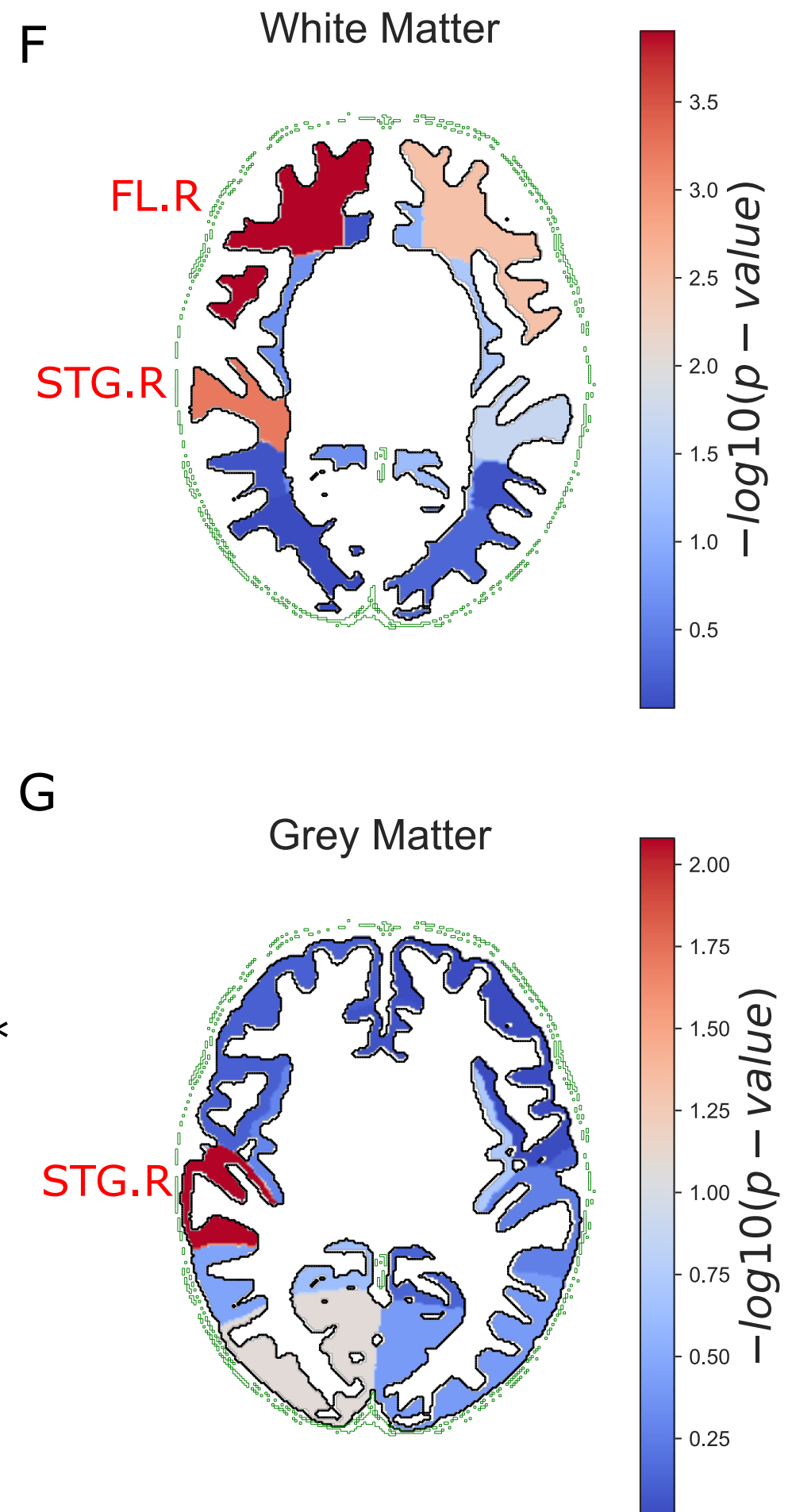

Supplement: Supplementary file 1 — Supplementary Figure 1 [file 41398_2023_2413_MOESM1_ESM.pdf]
