# Supplementary material for: Effect of schizophrenia common variants on infant brain volumes: cross-sectional study in 207 term neonates in developing Human Connectome Project
Source: Transl Psychiatry. 2023 Apr 10;13:121. doi: 10.1038/s41398-023-02413-6 (PMC10085987; doi:10.1038/s41398-023-02413-6)
Supplement: Supplementary file 2 — Supplementary Figure 1 legend [file 41398_2023_2413_MOESM2_ESM.docx]

**Supplementary Figure 1.** Visualisation of the significant association between the schizophrenia PRS and the regions of interest in the combined European and Asian ancestry cohort (n=257). A-D: Scatter plots of infant brain volume against Schizophrenia PRS at P_T_ = 0.001. Brain volumes have been adjusted for GA, PMA, sex and ICV. PRS have been adjusted for the first 5 ancestral principal components. Axes are standardised to zero mean and unit variance. The shaded area denotes the confidence interval E: Bar plot of brain volume variance R^2^ explained by PRS, calculated as the difference between the R^2^ of the full model with PRS as a covariate and that of the null model without the PRS as a covariate. * - nominal significant result; ** - result surviving multiple testing correction. F-G: Visualisation of the significant associations between the PRS at P_T_ = 0.001 and brain volumes of white (F) and grey matter (G) regions of interests. Colour bar denotes the range of –log_10_(p-value). (FL.R- right frontal lobe, STG.R- right superior temporal gyrus).
