# Supplementary material for: Effect of schizophrenia common variants on infant brain volumes: cross-sectional study in 207 term neonates in developing Human Connectome Project
Source: Transl Psychiatry. 2023 Apr 10;13:121. doi: 10.1038/s41398-023-02413-6 (PMC10085987; doi:10.1038/s41398-023-02413-6)
Supplement: Supplementary file 3 — Supplementary Information 1 [file 41398_2023_2413_MOESM3_ESM.docx]

**Supplementary information 1**

**Examination of family risk of psychiatric disorders and schizophrenia PRS**

Here, we identified 6 mental health questions relevant to family risk of psychiatric disorders, which were asked to the mother and father of each individual separately as follows:

1) “Have you ever been treated for a mental health problem?”,

2) “Have you ever been under psychiatric services?”,

3) “Have you ever been admitted to hospital for a mental health problem?”,

4) “Do you have a history of ADHD, bipolar disease, depression, autism, or schizophrenia?”,

5) “Have you or your child's biological father ever been diagnosed with ADHD or autism?”,

6) “Does anyone in your close family have a history of ADHD, bipolar disease, depression, autism, or schizophrenia?”.

If any of the questions were answered as “Yes”, the participant was classified as having family risk of psychiatric disorder. If at least one answer was recorded as “No” and all other answers were left missing, then the participant was classified as “not having family risk of psychiatric disorder”. If all answers were missing, the participant were excluded from this analysis.

Of the 207 participants in the European cohort, 71 (34%) were classified as having family risk, 79 (38%) were classified as not having family risk, and 57 (28%) were removed from the analysis. Of the 257 participants in the European and Asian cohort, 80 (31%) were classified as having family risk, 96 (37%) were classified as not having family risk, and 81 (32%) were removed from the analysis. No correlation between PRS and family risk status for either cohort was observed (European cohort: r = -0.018; pval = 0.829, Mixed cohort: r=0.035; pval = 0.648).
